# Magmaris bioresorbable magnesium scaffold in acute coronary syndromes: a systematic review and meta-analysis

**DOI:** 10.3389/fcvm.2026.1834712

**Published:** 2026-05-20

**Authors:** Simon Wölbert, Pablo F. F. Escrihuela-Branz, Stephanie Gladys Kühne, Andrea Patrignani, Mauro Chiarito, Jan Torzewski, Philip W. J. Raake, Dario Bongiovanni

**Affiliations:** 1 Department of Internal Medicine I, Cardiology, University Hospital Augsburg, University of Augsburg, Augsburg, Germany; 2 Department of Biomedical Sciences, Humanitas University, Pieve Emanuele, Milan, Italy; 3 Department of Cardiovascular Medicine, Humanitas Clinical and Research Center IRCCS and Humanitas University, Rozzano, Milano, Italy; 4 Cardiovascular Center Oberallgaeu-Kempten, Kempten, Germany

**Keywords:** acute coronary syndrome, bioresorbable scaffold, coronary artery disease, drug-eluting stent, magmaris, target-lesion failure

## Abstract

**Background:**

Bioresorbable scaffolds aim to mitigate long-term complications of drug-eluting stents (DES). The bioresorbable magnesium scaffold Magmaris is an alternative, but its role in acute coronary syndrome (ACS) remains insufficiently studied. Therefore, we aimed to evaluate safety and efficacy of Magmaris in ACS and compare outcomes with DES.

**Methods:**

We systematically searched PubMed, Embase and Cochrane Central Register of Controlled Trials for randomized and observational studies reporting outcomes of Magmaris in ACS. Primary outcome was target-lesion failure (TLF) defined as composite of cardiac death (CD), target-vessel myocardial infarction (TV-MI) and target-lesion revascularization (TLR) at one year. Secondary outcomes included scaffold/stent thrombosis (ST) and procedural success. Single-arm pooled event rates and pairwise comparisons were estimated using generalized linear mixed-effects models (GLMM).

**Results:**

We included four studies (*n* = 622 patients; 322 Magmaris, 300 DES) for pairwise analysis. At one year, no statistically significant difference in TLF was observed between Magmaris and DES (7.5% vs. 5.3%, OR 1.53, 95% CI 0.79–2.97; *p* = 0.21; *I*^2^ = 54.7%), although confidence intervals were wide. TLR was significantly higher with Magmaris (6.8% vs. 3.0%, OR 2.63, 95% CI 1.17–5.90; *p* = 0.019; *I*^2^ = 10.7%), while TV-MI, CD and ST did not differ significantly between groups. Twelve studies (*n* = 1,391 patients) contributed single-arm analyses showing pooled 1-year incidence of TLF 4.97%, TLR 4.16% and ST 0.83%. Procedural success was 98.49% (95% CI 97.21–99.18; *I*^2^ = 35.4%).

**Conclusion:**

In ACS, Magmaris was associated with low observed ST rates, nevertheless, comparative safety and efficacy vs. DES remain uncertain due to limited and heterogeneous evidence. Higher TLR rates with Magmaris suggest that further technical improvements in next-generation magnesium scaffolds are needed before this strategy can be considered a robust alternative in ACS.

## 1 Introduction

Percutaneous coronary intervention (PCI) with contemporary drug-eluting stents (DES) is considered gold standard in patients presenting with acute coronary syndrome (ACS) offering favorable short- and long-term outcomes with low rates of target-lesion failure (TLF). However, in the last two decades limitations of drug-eluting stents have become apparent. Systematic reviews and meta-analyses of randomized controlled trials have revealed an ongoing risk of 1%–2% for late and very-late stent thrombosis (1, 2). These complications can be partially attributed to the presence of a permanent foreign body in coronary arteries leading to chronic inflammation and neoatherosclerosis (3). The “*leave nothing behind”* strategy aims to overcome these limitations using bioresorbable vascular scaffolds (BVS). Since the first implantation almost 25 years ago (4), several large randomized trials investigated BVS outcomes compared to DES. The most studied device, the ABSORB BVS, was withdrawn from the market in 2017 due to increased risk of scaffold thrombosis (ST) during the resorption period (5). However, evidence from poly-L-lactic-acid (PLLA) based BVS implantation in patients presenting with ACS showed promising results (6, 7). In 2016, a new generation of magnesium-based fully bioresorbable scaffold (BRS), Magmaris, has received CE mark and has since shown sustained safety and efficacy for the treatment of *de novo* coronary artery disease (8, 9). Magmaris offers increased radial strength, decreased thrombogenicity and a shorter period of bioresorption in contrast to PLLA based BVS (10, 11). ACS typically occurs in younger patients and is driven by soft plaque rupture with a high thrombus burden, pathophysiologic features that conceptually align with the intended advantages of bioresorbable coronary scaffolds (12). Understanding the safety and efficacy of Magmaris in ACS is clinically relevant to guide further development of magnesium-based scaffold technologies. Prospective registries and subgroup analyses suggested that Magmaris represents a feasible and safe interventional option for the treatment of *de novo* coronary lesions in this high-risk cohort. However, available data remain fragmented and lack structured quantitative evaluation specifically in ACS. To address this gap, we conducted the first systematic review and meta-analysis including all clinical evidence available on Magmaris compared to DES implantation in ACS patients. Additionally, we performed a single-arm analysis and meta-regression to better understand potential confounding factors.

## 2 Methods

The review was performed and reported in accordance with the Preferred Reporting Items for Systematic Reviews and Meta-Analyses (PRISMA) guidelines and the Cochrane Collaboration Handbook for Systematic Reviews of Interventions (13, 14). The study protocol was prospectively registered in the International Prospective Register of Systematic Reviews (PROSPERO; CRD42024558675).

### 2.1 Eligibility criteria

We included studies fulfilling the following criteria (1): enrolled patients with ACS (2) reported revascularization with at least one Magmaris scaffold (3) reported at least one outcome of interest, and (4) had randomized controlled or observational design. Acute coronary syndrome comprised unstable angina, non-ST-elevation acute coronary syndrome (NSTE-ACS) and ST-elevation myocardial infarction (STEMI).

We excluded studies that: (1) included only patients with chronic coronary syndrome or did not report separately on ACS outcomes; (2) included patients treated with any other scaffold other than Magmaris (e.g., ABSORB BVS). Furthermore, conference abstracts, case reports and unpublished data were excluded.

### 2.2 Search strategy and data extraction

A comprehensive literature search was performed in PubMed, Embase (via Ovid) and the Cochrane Central Register of Controlled Trials from database inception to September 11th, 2025. Search strategies combined controlled vocabulary (MeSH and Emtree terms) with free-text keywords as follows: *bioresorbable magnesium scaffold (Magmaris, DREAMS2G, “bioresorbable magnesium scaffold”), acute coronary syndrome (“ST-elevation myocardial infarction”, “non-ST-elevation myocardial infarction”), coronary artery disease, stent, drug-eluting stent, absorbable implant*. Reference lists of eligible publications and reviews were screened to identify additional studies (backward snowballing). All searches and study selections were conducted independently by two reviewers (S.W., P.F.F.EB) in accordance with the PRISMA guidelines and subsequently extracted in prespecified data tables. Baseline clinical and procedural characteristics included: subtype of ACS, age, sex, diabetes mellitus, prior myocardial infarction (MI), predilation, postdilation, lesion complexity, location of culprit lesion, lesion length. Studies were also screened for procedure-related optimization characteristics, including adherence to the 4P implantation strategy, intravascular imaging, and lesion preparation/postdilation, as these factors are recommended by expert consensus (15). Disagreements were solved by consulting a third reviewer (D.B.). Detailed search strategies for individual databases are provided in Supplemental material search strategies***.***

### 2.3 Quality assessment

The methodological quality of single-arm studies was critically appraised using the Joanna Briggs Institute (JBI) Critical Appraisal Checklist for Case Series which comprises ten domains addressing study selection, measurement validity, outcome reporting and statistical analysis (16). Particular emphasis was placed on critical domains (clear eligibility criteria, consecutive and complete inclusion, adequacy of follow-up and appropriateness of statistical methods). Studies fulfilling at least eight domains without a critical failure were judged as low risk of bias, those with moderate limitations but no critical failure as some concerns and those with one or more critical failures as high risk of bias. Risk of bias in non-randomized studies was evaluated with ROBINS-I tool and risk of bias in randomized controlled trials was assessed using Cochrane Risk of Bias 2 (RoB2) tool (17, 18). Certainty of evidence for primary outcomes was evaluated using the Grading of Recommendations Assessment, Development and Evaluation (GRADE) approach, considering risk of bias, inconsistency, indirectness, imprecision, and publication bias (19). Two reviewers (S.W., P.F.F.EB.) independently performed risk of bias and certainty of evidence assessment. Disagreements were solved by consulting a third reviewer (D.B.).

### 2.4 Outcome measures

The primary outcome was target-lesion failure, defined as a composite of cardiac death (CD), target-vessel myocardial infarction (TV-MI) and clinically-driven target-lesion revascularization (TLR), according to Academic Research Consortium-2 (ARC-2) criteria (20). Secondary outcomes comprised the individual components of TLF, definite or probable scaffold thrombosis, procedural success, all-cause mortality (ACM). When studies reported endpoints using earlier ARC-1 definitions (21), these were extracted as originally defined. Outcomes were analyzed at 1 year and, when available, at 2 years of follow-up.

### 2.5 Statistical analysis

For single-arm analyses, pooled event rates and 95% CIs were derived using a generalized linear mixed-effects model (GLMM). This approach was chosen due to its generally superior performance compared with conventional two-step methods for meta-analyses of proportions (22). Heterogeneity among studies was assessed using *I*^2^ statistic and the Wald test for heterogeneity (23, 24). Additionally, for TLF and TLR single-arm analyses, 95% prediction intervals (PI) were calculated to complement pooled estimates and better reflect between-study variability.

To compare clinical outcomes between Magmaris and contemporary drug-eluting stents, pairwise meta-analyses were performed using the GLMM approach (25). For these analyses, odds ratios with 95% CIs were calculated and *p*-values were computed to assess statistically significant differences between the two groups.

Sensitivity analyses were performed using the leave-one-out method (LOO) for primary endpoints to assess the influence of individual studies on the pooled effect estimates. Study-level moderators were analyzed by means of a meta-regression to investigate their potential impact on logit event proportions (26).

A *p*-value ≤ 0.05 was considered statistically significant. All analyses were conducted using R version 4.3.3 with the meta package.

## 3 Results

### 3.1 Study selection and baseline characteristics

**Figure 1:**
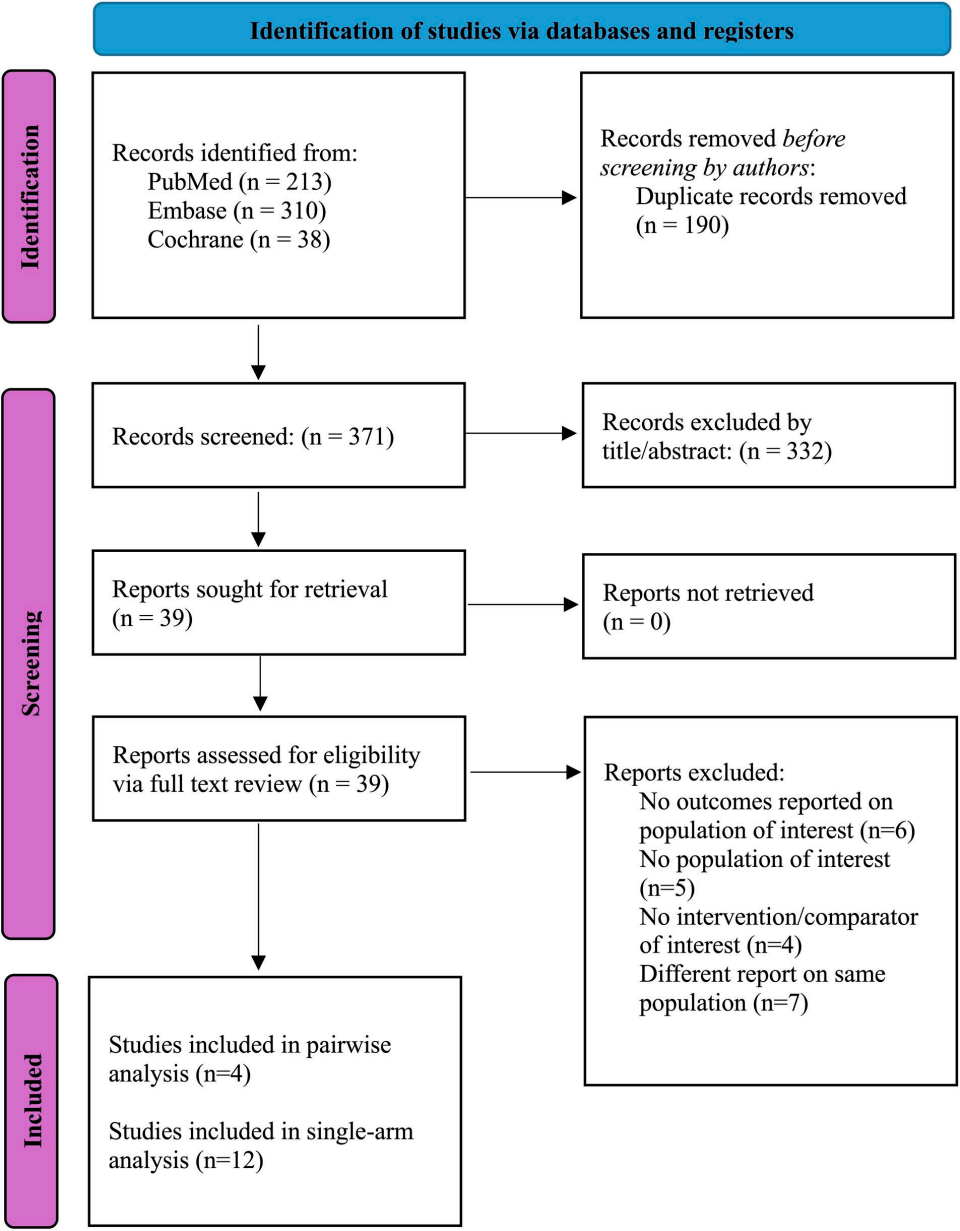
PRISMA 2020 flow diagram for new systematic reviews which included searches of databases and registers only.

The initial search identified 561 records. After removal of duplicates and exclusion of ineligible studies, 36 articles remained and were assessed according to predefined inclusion criteria. For the pairwise analysis, four studies were included, comprising 622 patients (322 Magmaris, 52%; 300 DES, 48%) from two randomized controlled trials, one propensity score–matched study and one observational study (27–30). For single-arm analysis at 1-year, twelve studies were included, encompassing a total of 1,391 patients (Figure 1) (8, 9, 27–36).

Across included studies, the mean age of patients ranged from 48.5 ± 10.0 to 66.3 ± 8.9 years, with a pooled unweighted average of approximately 58 years. In all studies, at least 64% of patients were male. The culprit lesion was most frequently located in the left anterior descending artery, with a median proportion of 50% (range 41%–66%, Supplementary Table S1, S2). Procedure-related optimization characteristics varied considerably across the included studies and are summarized in Supplementary Table S3. Among studies reporting these data, 4P adherence ranged from 66.7% to 100%, and intracoronary imaging use ranged from 8.3% to 100%, whereas predilation and postdilation were generally frequent but not uniformly documented. When reported, dual antiplatelet therapy (DAPT) after Magmaris implantation was generally intended for 12 months, although the specific antiplatelet regimen and its reporting varied across studies.

### 3.2 Primary and secondary outcomes

**Figure 2:**
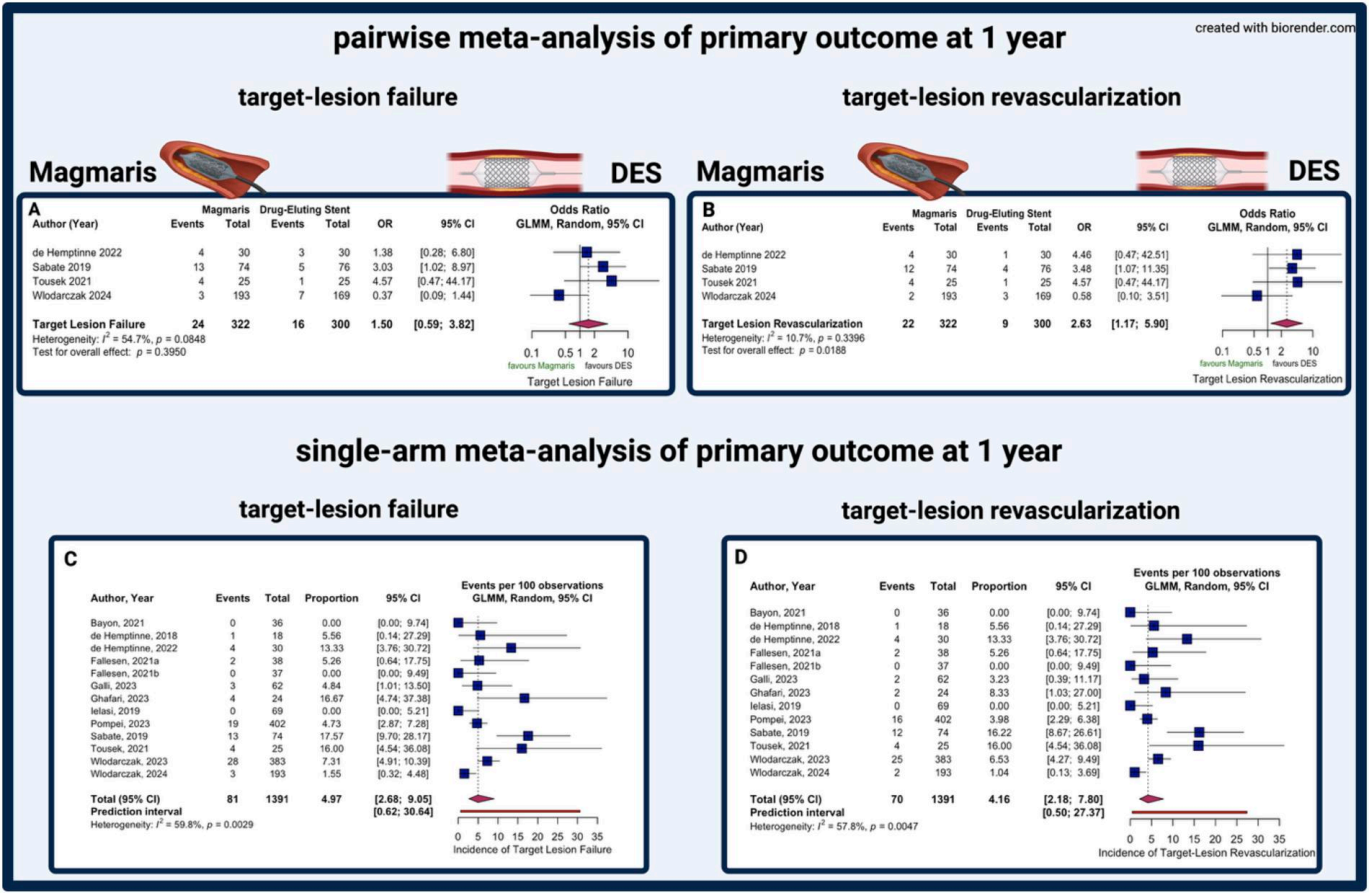
Pairwise and single-arm meta-analyses of 1-year target lesion failure and target lesion revascularization. Panels A and B display results of pairwise meta-analyses comparing Magmaris with drug-eluting stents for 1-year target-lesion failure **(A)** and 1-year target-lesion revascularization **(B)** Panels C and D show single-arm meta-analyses summarizing 1-year event rates for target lesion failure **(C)** and target lesion revascularization **(D)** CI, confidence interval; DES, drug-eluting stent; GLMM, generalized linear mixed-effects model; OR, odds ratio.

**Figure 3:**
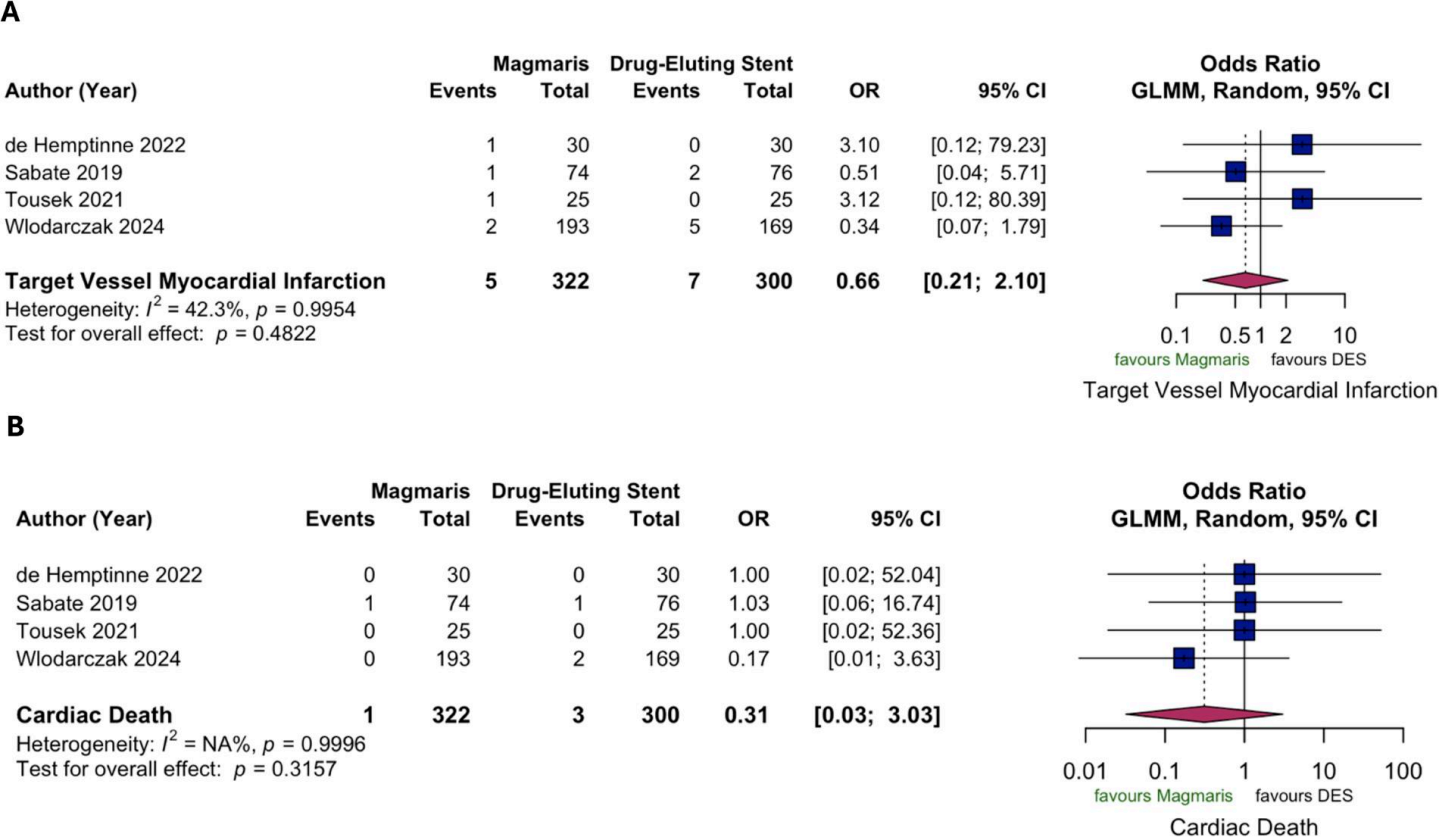
Pooled results of further individual target-lesion failure components (pairwise meta-analysis) at one year follow-up. **(A)** Pooled odds ratio for target-vessel myocardial infarction; **(B)** Pooled odds ratio for cardiac death. CI, confidence interval; DES, drug-eluting stent; GLMM, generalized linear mixed-effects model; OR, odds ratio.

At 1-year follow-up, no statistically significant difference was observed in TLF between Magmaris and DES (7.5% vs. 5.3%; OR 1.53, 95% CI 0.79–2.97; *p* = 0.21; Figure 2A). TLR was significantly higher in the Magmaris group (6.8% vs. 3.0%; OR 2.63, 95% CI 1.17–5.90; *p* = 0.019; *I*^2^ = 10.7%, Figure 2B), while no significant differences were observed in TV-MI (1.6% vs. 2.3%; OR 0.66, 95% CI 0.21–2.10; *p* = 0.48) and CD (0.3% vs. 1.0%; OR 0.31, 95% CI 0.03–3.03; *p* = 0.32; Figures 3A,B). Scaffold/stent thrombosis rates were 0.6% in the Magmaris group and 1.0% in the DES group (OR 0.67, 95% CI 0.11–4.09; *p* = 0.67; Supplementary Figure S1A). ACM rates were 0.9% and 0.3%, respectively (OR 2.88, 95% CI 0.30–27.92; *p* = 0.36; *I*^2^ = 42.3%, Supplementary Figure S1B). Heterogeneity across studies was substantial for TLF (*I*^2^ = 54%), moderate for TV-MI and CD (*I*^2^ = 42.3%) and ST (*I*^2^ = 44.8%), and low for TLR (*I*^2^ = 10.7%).

**Figure 4:**
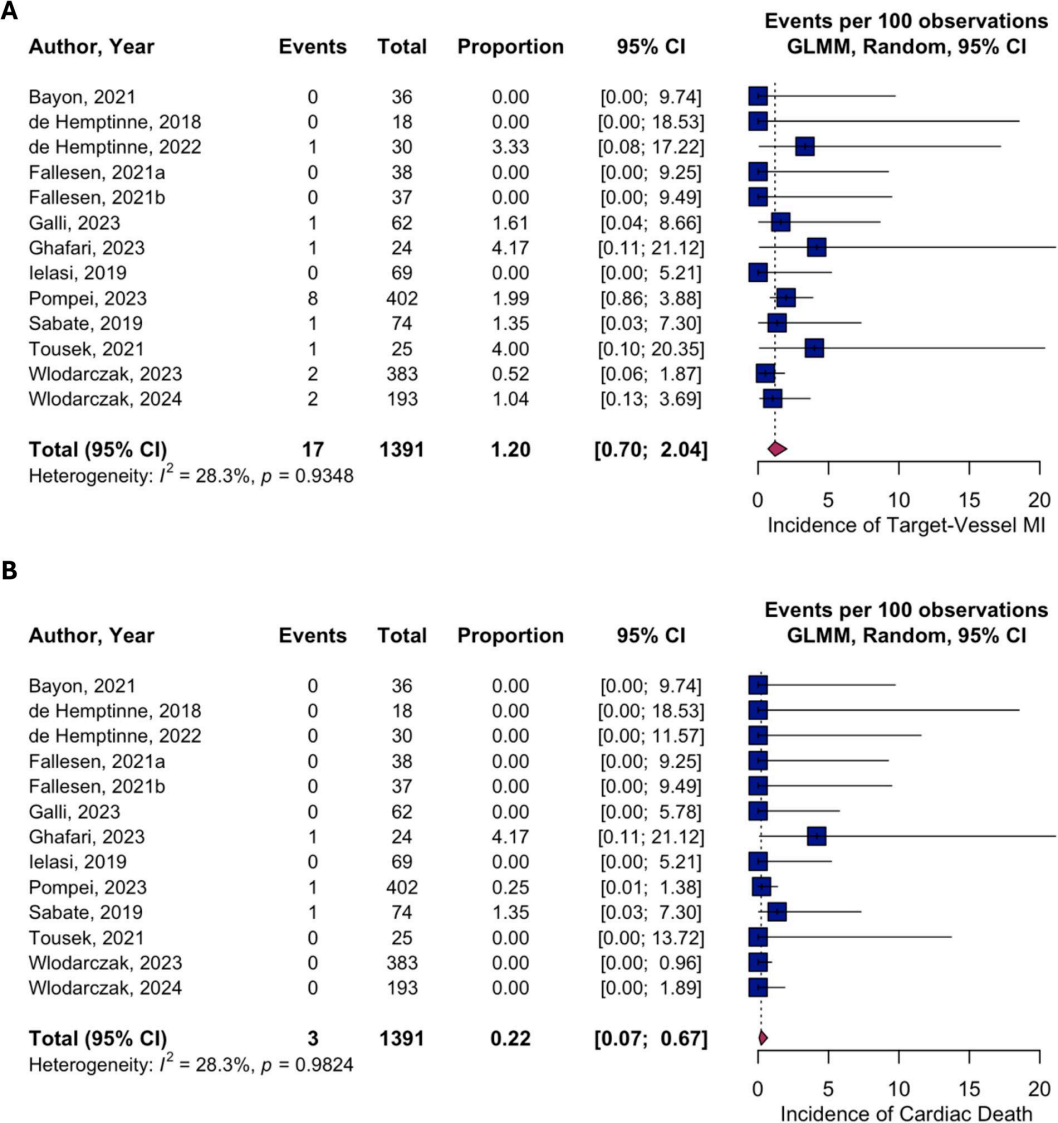
Pooled incidence of individual target-lesion failure components (single-arm meta-analysis). **(A)** Incidence of target-vessel myocardial-infarction; **(B)** Incidence of cardiac death. CI, confidence interval; GLMM, generalized linear mixed-effects model; MI, myocardial infarction.

In the single-arm meta-analysis, pooled 1-year incidence rates following Magmaris implantation were 4.97% for TLF (95% CI 2.68–9.05; PI 0.62–30.64; Figure 2C), 4.16% for TLR (95% CI 2.18–7.80; PI 0.50–27.37; Figure 2D), 1.20% for TV-MI (95% CI 0.70–2.04; *I*^2^ = 28.3%,), 0.22% for CD (95% CI 0.07–0.67; *I*^2^ = 28.3%, Figures 4A,B) and 0.83% for scaffold thrombosis (95% CI 0.43–1.60; *I*^2^ = 28.3%, Supplementary Figure S2A). Procedural success was consistently high across all studies (98.49%, 95% CI 97.21–99.18; *I*^2^ = 35.4%, Supplementary Figure S2B). ACM yielded an incidence of 0.62% (95% CI 0.31–1.23, I^2^ = 26.2%, Supplementary Figure S2C). Heterogeneity was substantial for TLF (*I*^2^ = 59.8%) and TLR (*I*^2^ = 57.8%), while it was low for TV-MI (*I*^2^ = 28.3%), scaffold thrombosis (*I*^2^ = 28.3%), cardiac death (*I*^2^ = 28.3%), and procedural success (*I*^2^ = 35.4%). Meta-regression analysis revealed that study-level NSTE-ACS proportion significantly influenced TLF rates, with each 10% increase in NSTE-ACS patients associated with a 16% reduction in TLF risk (OR 0.844, 95% CI 0.74–0.96; *p* = 0.010, Supplementary Figure S3A). In contrast, diabetes prevalence showed no significant association with TLF outcomes (OR 1.008, 95% CI 0.94–1.08; *p* = 0.829, Supplementary Figure S3B).

At 2-year follow-up, available in seven studies including a total of 818 patients, the pooled single-arm incidence rates were 9.08% (95% CI 6.40–12.73; *I*^2^ = 38.8%) for TLF, 7.58% (95% CI 5.95–9.60; *I*^2^ = 30.9%) for TLR, and 1.22% (95% CI 0.66–2.26; *I*^2^ = 0.0%) for scaffold thrombosis (Supplementary Figures S4A–C).

### 3.3 Sensitivity analysis

Leave-one-out analysis (LOO) generally confirmed robustness of our analysis (Supplementary Figures S5A,B). For TLF, the pooled estimate remained largely unaffected by sequential study exclusion (OR range 1.03–2.64), while TLR remained significantly higher for Magmaris (OR range 2.00–3.83; Supplementary Figure S5B). Collectively, these findings indicate that the observed TLR and TLF results are mostly consistent and stable across sensitivity analyses.

For single-arm analyses, LOO demonstrated stability of the pooled estimates. For TLR, the pooled event rate remained consistent between 3.65% and 5.0%; rates for TLF ranged between 4.39% and 5.88%, indicating that no single study unduly influenced the overall results (Supplementary Figures S6A,B).

### 3.4 Quality assessment

The overall methodological quality of the included studies was variable. Case series were predominantly rated as having *some concerns* according to the JBI checklist, while non-randomized comparative studies demonstrated a *serious risk of bias* in several domains when assessed with ROBINS-I (Supplementary Tables S4,S5). Among randomized controlled trials, one study was judged to be at *low risk*, and one at *some concerns* based on RoB2 evaluation (Supplementary Table S6). Overall, the certainty of evidence for key outcomes was considered moderate according to GRADE (Supplementary Table S7).

## 4 Discussion

To date, this systematic review and meta-analysis provides the most comprehensive synthesis of clinical data following implantation of the Magmaris bioresorbable magnesium scaffold in patients presenting with ACS. The principal findings are as follows: (1) In the pairwise analysis, no statistically significant differences were observed between Magmaris and DES for TLF, TV-MI, CD, and ST; however, these estimates were derived from a small and heterogeneous evidence base; (2) TLR occurred more frequently with Magmaris than with DES, indicating a higher need for repeat interventions during the first year. (3) Single-arm pooled analyses provided descriptive estimates of event rates with low incidences of ST (0.8%) and CD (0.2%) at one year, supporting an overall acceptable safety profile in an ACS population.

In the pairwise analysis, although no statistically significant difference was observed for TLF, this finding should be interpreted with caution and not as evidence of equivalence between Magmaris and DES, given the limited precision of the effect estimate and the wide confidence interval. This is further underscored by the point estimate, which numerically suggested a higher risk with Magmaris. Leave-one-out analyses revealed an important context to this finding: omission of the Wlodarczak et al. study rendered the difference in TLF statistically significant, suggesting that this cohort attenuated the overall effect estimate. A plausible explanation is the recognized experience of this study group in scaffold implantation, which may have resulted in more consistent lesion selection, implantation technique, and scaffold optimization, thereby contributing to comparatively favorable Magmaris outcomes in that cohort. Although this interpretation remains speculative, it may explain why this study appears to differ from the other comparative datasets. In contrast, Magmaris was associated with a significantly higher TLR rate than DES. One possible contributing factor is that several trials, including MAGSTEMI (27), Fallesen et al. (35) and Toušek et al. (28), implemented mandatory angiographic follow-up, a strategy known to increase detection of asymptomatic restenosis and clinically silent late lumen loss. Notably, leave-one-out analysis showed that the difference in TLR was no longer statistically significant after exclusion of MAGSTEMI, indicating that this finding, although directionally consistent, remained partly dependent on this study for statistical precision. At the same time, a plausible biological explanation also merits consideration: the lower radial strength and time-limited scaffolding of Magmaris, which are associated with greater late lumen loss and a higher likelihood for restenosis. Taken together, the excess TLR observed with Magmaris most likely reflects an efficacy limitation of the current platform, while differences in surveillance intensity may have influenced the magnitude of this effect. The pattern suggests that while the “*leave-nothing-behind”* concept can be realized without compromising early safety, the second-generation magnesium scaffold still shows limitations in long-term luminal preservation relative to contemporary DES. The observed odds ratio of 2.6 for TLR translates to an absolute risk increase of approximately 3%–4% at one year, which is clinically meaningful despite its seemingly modest magnitude. The consistent tendency toward higher repeat revascularization with Magmaris compared with contemporary DES is particularly relevant in the ACS setting where early luminal stability is critical. Nevertheless, this short-term disadvantage must be weighed against the potential long-term advantages of a fully bioresorbable scaffold, including the theoretical reduction of very late stent-related adverse events. Ultimately, whether this early reintervention trade-off is offset by durable long-term benefits remains an open question that future adequately powered randomized trials must clarify.

Different DES were used in trials comparing Magmaris with contemporary DES; specifically, Orsiro and Xience were used and Orsiro has been shown to be superior to Xience in terms of target-lesion failure (37, 38). This might reduce generalizability of our findings to all types of DES.

Beyond the paired analysis, our single-arm analysis summarizes the currently available clinical evidence of a magnesium-based BRS in ACS by providing pooled estimates of observed event rates. Within this descriptive framework, scaffold thrombosis remained infrequent at one and two years (0.83% and 1.22%, respectively), lower than the rates historically reported for first-generation PLLA scaffolds such as ABSORB for which 12-month thrombosis rates exceeded 4% in randomized trials (39); despite the higher-risk clinical profile of ACS patients. Of note, the persistently low scaffold-thrombosis rate at two years represents an interesting finding given the historical concerns surrounding bioresorbable scaffolds. This signal is biologically plausible given the faster, more homogeneous resorption of the magnesium alloy as well as its antithrombotic characteristics, thinner strut architecture, and reduced risk for late scaffold dismantling (11). The pooled incidences of TLF, approximately 5% at one year and 9% at two years, were broadly comparable to all-comers registry data such as BIOSOLVE-IV (9), suggesting that ACS patients treated with Magmaris do not experience disproportionately worse outcomes. These findings are reassuring, but they should be interpreted in the context of moderate heterogeneity and predominance of observational cohorts.

Interestingly, meta-regression analysis indicated an association between a higher proportion of NSTE-ACS patients and lower pooled TLF rates. This may be partially attributable to differences in plaque morphology: thrombus-rich STEMI lesions with large necrotic cores may benefit less from a scaffold that offers only short-term radial support, whereas NSTE-ACS presentations often involve more stable plaque characteristics. Differences in lesion substrate between ACS phenotypes may therefore contribute to this pattern, but the available data do not allow mechanistic conclusions. Moreover, in STEMI intervention, stent undersizing could be a more common feature, possibly leading to major issues in case of underexpanded BVS. Given the limited number of studies, the study-level nature of the analyses, potential collinearity with STEMI prevalence and risk for ecological bias, this finding should be interpreted with caution and viewed as hypothesis-generating rather than confirmatory.

Diabetes mellitus was not associated with worse outcomes in this meta-analysis (Supplementary Figure S3B). Although the limited spread of diabetes prevalence across included studies may have constrained the ability to detect a moderating effect, the absence of a detrimental signal is clinically relevant given the traditionally elevated restenosis risk in diabetic patients and suggests that Magmaris is not intrinsically disadvantaged in this subgroup when implanted with optimized technique (40).

The moderate heterogeneity observed for TLF and TLR (*I*^2^ ≈ 60%) in single-arm analyses appears largely attributable to clinical and methodological variability across studies, particularly differences in ACS subtype distribution, trial design (randomized vs. observational), and the use of mandatory angiographic follow-up in several RCTs. This is further reflected by the wide prediction intervals observed for the main single-arm outcomes. Procedure-related factors deserve particular attention in the context of bioresorbable scaffolds, as prior experience with scaffold technologies has shown that careful lesion preparation and implantation optimization are closely linked to clinical outcomes (41). In the present dataset, adherence to scaffold-specific implantation protocols and the use of intracoronary imaging were not uniform across studies. At one end of the spectrum, some cohorts reported highly standardized optimization strategies, including routine 4P-adherence in implantation and near-systematic imaging guidance (28, 35). In contrast, other cohorts reported only selective or discretionary use of imaging, for example Ghafari et al. with IVUS in 8.3% and Włodarczak et al., in which OCT/IVUS support was left to operator discretion within a 4P-oriented implantation strategy (9, 31). Intermediate levels of procedural standardization were also observed, as illustrated by Gutiérrez-Barrios et al., where OCT guidance was used in 68.9% and the full 4Ps protocol was documented in 66.7%, and by SHERPA-MAGIC, where predilatation, 1:1 sizing and post-dilatation were protocolized, while IVUS/OCT was strongly encouraged and used in more than half of cases (8, 42). Taken together, this heterogeneity in procedural optimization likely influenced event rates and could not be accounted for in a standardized fashion across the pooled analyses.

Although our primary outcome was based on ARC-2 criteria, endpoint definitions were not fully uniform across the included studies. In particular, several predominantly observational single-arm studies and registries relied on earlier ARC-based or other study-specific definitions, especially for myocardial infarction adjudication, which may have contributed to additional clinical heterogeneity (8, 9, 31–34, 42). The aforementioned trial-specific characteristics likely account for a substantial proportion of the between-study dispersion; however, heterogeneity could not be fully explained, indicating that additional unmeasured sources of variability may persist. This interpretation is consistent with the risk-of-bias (ROB) and GRADE assessments: although overall certainty of evidence was rated moderate, the comparative evidence base is limited to only four studies, and two of the non-randomized cohorts were judged to have serious ROB due to confounding and selection biases [de Hemptinne et al., (29); Wlodarczak et al., (9)]. Consequently, while the direction of the effect estimates is consistent, the strength of inference remains constrained by study quality, residual heterogeneity, and design differences.

The focus on 1-year outcomes is particularly relevant for magnesium-based scaffolds, as Magmaris undergoes rapid bioresorption with substantial loss of structural integrity within the first 6 months and near-complete resorption by approximately 12 months (28). This contrasts with earlier PLLA-based platforms such as Absorb, which exhibited prolonged resorption periods of up to 3 years and delayed emergence of safety concerns (43). Therefore, 1-year outcomes capture the critical period of scaffold resorption and vascular healing for magnesium-based devices, although longer-term follow-up remains important to fully assess durability of clinical outcomes.

The performance of DREAMS 2G provides an important proof of concept: although efficacy appeared inferior to contemporary DES, most notably with higher TLR, the low observed scaffold thrombosis rates suggest that fully bioresorbable magnesium platforms remain a viable concept in ACS. Clinically, this highlights both the promise and the current limitations of the technology: restenosis leading to increased need for repeat percutaneous coronary intervention remains the principal barrier to broader adoption, while, based on available data, the low observed ST rates support continued development. Next-generation scaffolds such as DREAMS 3G/Freesolve, featuring higher radial strength, thinner struts, and optimized drug release, are specifically engineered to address these shortcomings. The ongoing BIOMAG II randomized trial will be central in determining whether these refinements can close the efficacy gap without compromising safety (44). In the meantime, future studies should standardize not only implantation technique, but also adjunctive treatment strategies including DAPT and follow-up protocols, to allow more robust comparisons across scaffold-based PCI studies.

## 5 Limitations

This study-level meta-analysis was based on aggregated published data rather than individual patient data, which limits the ability to adjust for patient-level confounders and to explore interaction effects across clinically relevant subgroups (e.g., STEMI vs. NSTE-ACS, sex, or diabetes status) in a robust way. Second, heterogeneity in endpoint definitions, implantation protocols, and follow-up durations may have influenced the pooled estimates, although sensitivity analyses confirmed the robustness of the primary outcomes. Third, the number of comparative studies was small, reducing statistical power to detect differences in rare events such as scaffold thrombosis. Moreover, pooling randomized with non-randomized comparative designs may have introduced residual confounding and limited the internal validity of comparative inferences. In addition, comparator DES were limited to Orsiro, Xience and Ultimaster, which may restrict generalizability. Fourth, procedure-related factors, including intracoronary imaging and scaffold optimization strategies, were inconsistently reported across studies and could therefore not be evaluated in a standardized fashion. Furthermore, most included studies were conducted at medium- to high-volume PCI centers with experience in BRS implantation. While this reflects standard practice in device trials, generalizability to lower-volume centers or operators with less BRS experience may be limited. Another limitation is that the meta-regression analyses were based on a small number of studies and should be interpreted as exploratory, particularly given the risk of overfitting and ecological bias inherent to study-level analyses. Publication bias cannot be excluded. However, formal assessment was considered of limited interpretability given the small number of studies and the known limitations of funnel-plot asymmetry methods (45). Finally, given that Magmaris has been largely discontinued commercially, our findings mainly inform the development of next-generation magnesium scaffolds.

## 6 Conclusion

In this systematic review and meta-analysis, Magmaris was associated with low observed ST rates in ACS across short- and mid-term follow-up, whereas comparative safety and efficacy vs. DES remain uncertain as the available evidence is limited and heterogeneous. The higher TLR rates observed with Magmaris indicate a current efficacy limitation of the platform, underscoring the need for technical improvements in next-generation magnesium scaffolds currently undergoing clinical testing before this strategy can be considered a robust alternative in ACS.
